# Effects of vibration training combined with kinesio taping on delayed onset muscle soreness of athletes’ knee joints post-DOMS induction: a randomised controlled trial

**DOI:** 10.3389/fbioe.2025.1561309

**Published:** 2025-04-02

**Authors:** Liang Cheng, Yunfei Jiang, Benxiang He

**Affiliations:** ^1^ Sichuan Academy of Chinese Medicine Sciences, Chengdu, China; ^2^ School of Sports Medicine and Health, Chengdu Sport University, Chengdu, China; ^3^ Human Movement Science, Sichuan Sports College, Chengdu, China

**Keywords:** delayed onset muscle soreness, vibration training, kinesio taping, pain, muscle strength

## Abstract

**Objective:**

This study aimed to investigate the effects of vibration training combined with kinesio taping on delayed onset muscle soreness (DOMS) in athletes.

**Methods:**

Forty-five athletes were randomly divided into the vibration group (n = 11), kinesio group (n = 11), combined group (n = 12) and control group (n = 11) to establish DOMS models of the knee. The visual analogue scale (VAS), peak torque of knee extension (60°/s), serum interleukin-6 (IL-6) and creatine kinase (CK) were measured at baseline, immediately and 24, 48 and 72 h later.

**Results:**

In terms of VAS, the combined group was lower than the control group (immediately and 24 h, *p* < 0.05). The vibration group, kinesio group and combined group had lower VAS scores than the control group, and the vibration group and kinesio group had higher VAS scores than the combined group (48 and 72 h, *p* < 0.001). The IL-6 and CK levels of the vibration group, kinesio group and combined group were lower than those of the control group (immediately, 24 and 48 h, *p* < 0.05), but those of the vibration group and kinesio group were higher than that of the combined group (24 h, *p* < 0.05). The peak torque of knee extension in the combined group was higher than that in the control group (24 h, *p* < 0.05).

**Conclusion:**

Vibration training and kinesio taping can alleviate muscle pain and reduce serum IL-6 and CK concentrations and muscle strength loss caused by DOMS in athletes to varying degrees, with similar effects. Compared with single intervention, combined intervention is more effective in reducing the inflammatory response and muscle micro-injury and in reducing muscle pain and muscle strength loss. These findings provide evidence-based strategies for optimizing recovery protocols in athletic training programs.

## Introduction

Delayed onset muscle soreness (DOMS) often occurs after unaccustomed sports or movements, typically within 8–24 h post - intense eccentric exercise, peaking at 72 h and recovering in about 1 week (Cheng et al., 2022a). The knee joint, crucial for weight transmission and force conversion, shows varying DOMS severity across sports. In sports like running, football and basketball with high knee impact and load, DOMS is more pronounced (Keriven et al., 2023; Sonkodi et al., 2023).

Appropriate vibration training can alleviate DOMS in ordinary people. It significantly reduces serum CK concentration 48 h after training in ordinary young women with elbow DOMS (50 Hz, 5 min) (Imtiyaz et al., 2014). It also increases MVIC at 72 h after inducing DOMS in ordinary young men/women with radial extensor carpi ulnaris DOMS (20 Hz, 2 min) (Koh et al., 2013). Vibration training reduces VAS scores immediately and at 24 and 48 h in ordinary male college students with knee DOMS (20–45 Hz, 10 min) (Wheeler and Jacobson, 2013), but increases immediate VAS in those with elbow DOMS (20 Hz, 30 min) (Lau and Nosaka, 2011). Our prior study found that 25 Hz vibration training was ineffective, while 50 Hz could lessen muscle pain and strength loss due to DOMS in athletic athletes, and decrease serum IL - 6 and CK concentrations (Cheng et al., 2022a). Vibration training decreases DOMS following eccentric exercise in elite hockey players (Akehurst et al., 2021), and reduces muscle soreness perception in elite athletes after eccentric exercises (Iodice et al., 2019).

Kinesio taping benefits ordinary people with DOMS. It can reduce biceps soreness and accelerate MVIC recovery in young men when applied before DOMS modeling (Lee et al., 2015). It lowers the cold sensation and pain thresholds of the biceps (DOMS) in ordinary men at 24 h and VAS scores immediately and at 24 and 48 h (Bae et al., 2014). It also reduces the pain of the rectus femoris and hamstring (DOMS) in young women at 24 and 168 h (Haksever et al., 2016). For athletes, kinesio taping can reduce DOMS - induced muscle pain but is less effective at reducing skeletal muscle micro - injury (Song and Yang, 2021). It was found more effective than sham taping in reducing pain intensity and delaying fatigue - inducing time in calf muscles with acute - onset muscle soreness among endurance athletes (Malhotra et al., 2022). A study showed that combining vibration training and kinesio taping further alleviates muscle pain and strength loss due to DOMS in badminton college students, though blood data were not collected (Que, 2023). The synergistic potential of combining vibration training and kinesio taping may arise from their complementary mechanisms: vibration training enhances blood circulation and neuromuscular activation, while kinesio taping facilitates lymphatic drainage and proprioceptive feedback. This multimodal approach could theoretically accelerate recovery processes more effectively than isolated interventions. Despite evidence of these interventions’ potential to alleviate DOMS in the general population (Imtiyaz et al., 2014; Koh et al., 2013; Wheeler and Jacobson, 2013; Lau and Nosaka, 2011; Lee et al., 2015; Bae et al., 2014; Haksever et al., 2016; Song and Yang, 2021), research on athletes is limited, and combined intervention studies are scarce. Given athletes’ high performance and recovery demands during training and competition (Cheng and Jiang, 2020), and DOMS’s potential negative impact on them, this study aims to fill this research gap and provide effective DOMS management strategies for athletes.

This study examines the individual and combined effects of vibration training and kinesio taping on managing DOMS in athletes. It aims to provide new insights for sports science and help develop customized training and recovery programs, enhancing athletes’ performance and reducing injury risk. We hypothesize that compared to single-modality interventions, the combined application of vibration training and kinesio taping will demonstrate significantly greater reductions in pain perception (VAS scores), inflammatory markers (IL-6, CK), and muscle strength loss (peak torque) at 24–72 h post-intervention.

## Materials and methods

### Participants

This study is registered with ClinicalTrials.gov (NCT06535321). This study was approved by the Human Experiment Ethics Committee of Chengdu Sport University (No: 2103). Trial Date: May 2024. This study referred to our previous research on the effect of vibration training on DOMS in athletes (Cheng et al., 2022a) and the research on the effect of kinesio taping on DOMS in athletes (Bae et al., 2014; Song and Yang, 2021). Considering the experimental design of four groups and five measurements per group, about 5% of the samples were lost. The VAS scores, IL-6 levels and CK levels as indicators of DOMS exhibited high sensitivity and specificity. Using G-power software, the effect size of 0.3 was selected (Cheng et al., 2022a; Bae et al., 2014; Song and Yang, 2021). Power was set at 0.8, and the significance level was set at 0.05. At least 40 samples were needed for calculation.

Track and field athletes from Sichuan Province, China, were recruited during the off-season in 2024. Inclusion Criteria: athletes who performed regular training, ranked in the top three in a provincial competition and trained at least 5 days per week, with a minimum of 4 h each day; this training encompassed, but was not limited to, strength training, endurance training, skill drills and competitive preparation; this study was in line with the Declaration of Helsinki, and the participants were aware of the study intention and signed informed consent. Exclusion Criteria: lower limb joint injury and systematic exercise training in the last week. A total of 45 participants (male/female: 26/19) meeting the criteria were recruited, and no sample loss was observed throughout the experimental process. The participants were divided into the vibration group (n = 11), kinesio group (n = 11), combined group (vibration training + kinesio taping, n = 12) and control group (n = 11) by digital random grouping. No statistically in age, height and body mass amongst groups were observed (Table 1).

**TABLE 1:** Participant information.

Group	Male/Female	Age (y)	Height (cm)	Body mass (kg)
Vibration group (n = 11)	7/4	20.3 ± 1.5	174.0 ± 6.3	66.8 ± 8.6
Kinesio group (n = 11)	6/5	20.2 ± 1.1	173.8 ± 6.9	66.7 ± 7.9
Combined group (n = 12)	7/5	20.4 ± 1.3	173.5 ± 5.7	66.6 ± 8.3
Control group (n = 11)	6/5	20.0 ± 1.2	174.2 ± 5.8	66.9 ± 7.6

### DOMS modelling

Referring to our previous experimental design for DOMS modelling of athletes’ knee joints (Cheng et al., 2022a), we performed a detailed oxygen uptake test (Cortex Cardiopulmonary Function Test System, Model: Metalyzer 3B, Germany) on all participants at 1 week before DOMS modelling. A detailed record was made 1 week after DOMS modelling. Participants performed downhill running on a treadmill (ICON, Model: NETL28717, United States; gradient of −10°, five groups, 8 min/group, 2 min of flat walking between groups). According to the maximum oxygen uptake, exercise intensity was matched for each participant, ensuring that all participants included had similar aerobic capacity and could maintain similar intensity during downhill running to complete DOMS modelling. Participants wore a Polar watch (Finland, Model: M430) throughout the course to monitor their heart rate, ensuring that 80% of maximum heart rate was maintained during downhill running. If the heart rate was too low or too high, the treadmill speed was adjusted appropriately. Within 72 h after DOMS modelling, we followed up with the participants to ensure that they did not undergo other training activities or treatments.

The following interventions were performed on each group in this study. For the vibration group, vibration training was performed immediately after DOMS modelling. For the kinesio group, Y-shaped kinesio taping was performed 30 min before DOMS modelling. After modelling, when the vibration platform is closed, all participants of the kinesio group and control group completed the same action as vibration training. For the combined group, the vibration group and kinesio group interventions were performed simultaneously.

### Vibration training

On the basis of our previous experimental design and research results of vibration training intervention for athletes, a vibration training intervention with a frequency of 50 Hz, an amplitude of 3 mm and a duration of 10 min was selected (Cheng et al., 2022a). Using a Power Plate vibrating platform (Model: Power Plate pro5TM, United States), vibration training was performed on the participants of the vibration group and combined group who had completed DOMS modelling under the guidance of the experimenter. The participants performed a half squat (3 min) and a lunge pull (3.5 min on each side) on the vibrating platform (Cheng et al., 2022a). The kinesio group and control group participants performed the same number of sets and times as the vibration group and combined group participants when the vibrator was turned off. Vibration training was performed only once, immediately after DOMS molding.

### Kinesio taping

All taping procedures were performed by two certified physiotherapists following standardized protocols. Y-shaped kinesio taping was performed 30 min before DOMS modelling (Song and Yang, 2021). The participants took a sitting position, keeping their knees bent at 90°. Professional physiotherapists used 5 cm × 5 m intramuscular adhesive tape (LP Support, United States, size: 5 cm × 5 m, colour: blue) to tape the bilateral vastus medialis, vastus lateralis and rectus femoris in a ‘Y’ shape (stretched to 125% of the original length) (Que, 2023). To avoid the detachment of the muscle tape, we re-taped the same operation at 24, 48 and 72 h. During DOMS modelling, most of the participants did not lose the muscle tape, but some participants lost part of the tape, which was re-taped immediately.

This study evaluated the VAS scores, serum CK and IL-6 levels and peak torque of knee extension (60°/s) before DOMS modelling (baseline and at 24 h before DOMS modelling) and after vibration training (immediately), 24, 48 and 72 h of the participants in the four groups.

### VAS testing

The pain degree of the participants at the DOMS site was evaluated by VAS. The participants were asked to draw a vertical line on a 10 cm straight line (0 cm was painless, and 10 cm was the most painful, scored 0–10. The left and right knee joints were drawn once each, and the mean was processed (Chang S. et al., 2024).

### Serum IL-6 and CK testing

Our previous methods provide further details (Cheng et al., 2022a). About 3 mL of venous blood was collected from the participants, and the CK concentration of the participants at five time points was detected by using an RT-9600 automatic biochemical analyser (kit provided by Shanghai Lanxing Biotechnology Co., Ltd.). Serum IL-6 was detected by enzyme-linked immunosorbent assay (kit provided by Shanghai Varan Biotechnology Co., Ltd.).

### Isokinetic knee testing

An IsoMed 2000 isokinetic tester from Germany was used to perform 60°/s (three times) extension test on the participants’ knee. The participants were in a sitting position, and the trunk and hip joints were fixed with a wide binding. The knee joint extension mode was selected with the joint activity of 80°, and the analytical index was peak torque; the test data were averaged on the left and right sides (Cheng et al., 2022b).

### Statistical analysis

The mean ± standard deviation of the five test data of the four groups of participants was processed by SPSS 20.0. Two-way repeated measures ANOVA was used to analyse the main effects of group and time and determine whether an interaction occurred between group (4) and time (5). If an interaction was found between group and time, the differences at different time points within the group were analysed by repeated measures ANOVA. If time had a main effect, the differences at different time points were compared *post hoc*. If group had a main effect, the differences at the same time point between groups were compared *post hoc* (Cheng et al., 2022a; Cheng et al., 2024; Chang S. W. et al., 2024). The *post hoc* comparison was adjusted by Bonferroni to ensure that the overall type I rate of each ANOVA was not greater than 0.05. The significance level was α = 0.05.

## Result

**FIGURE 1:**
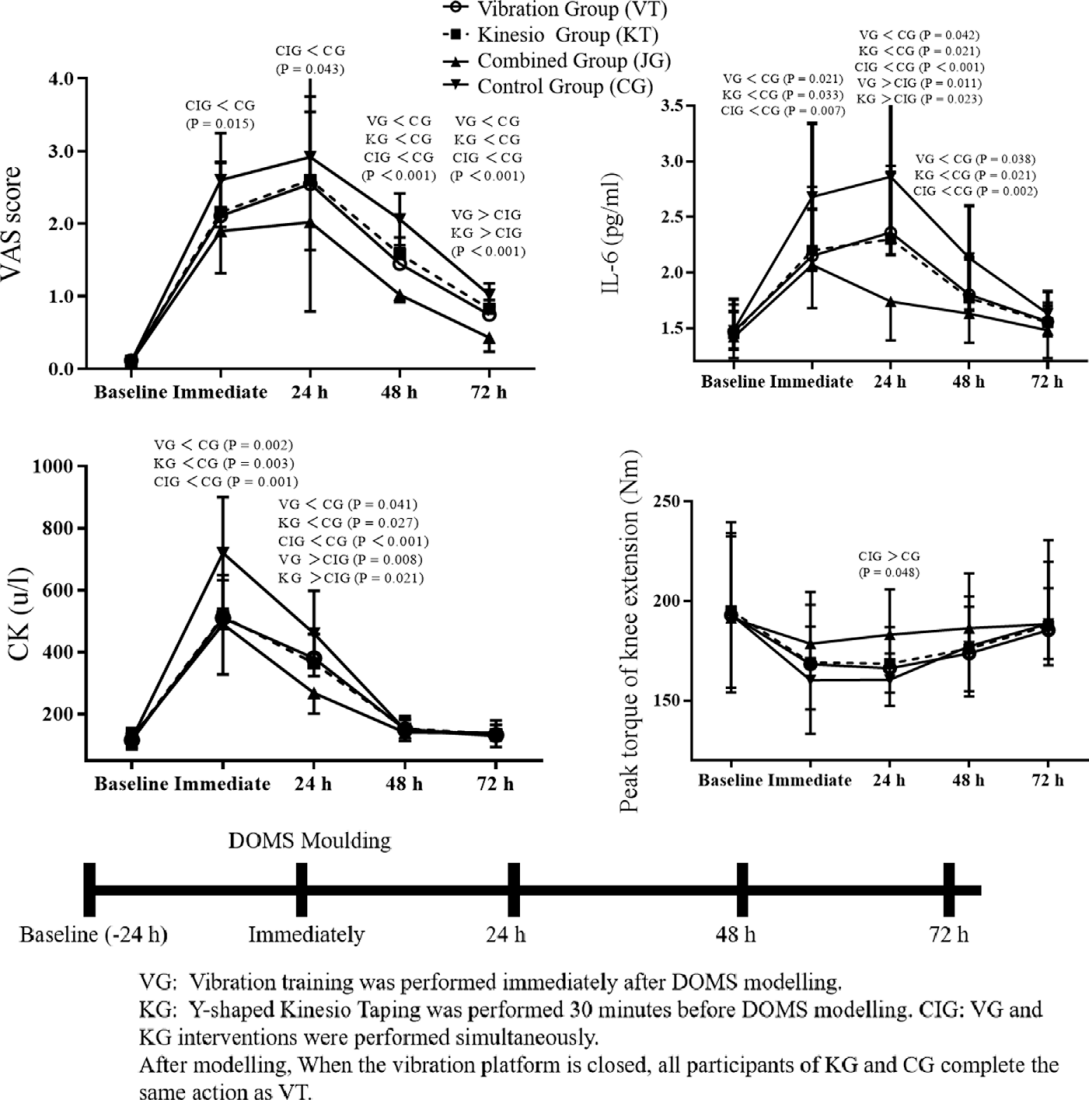
Changes in indicators of participants at different time points.

The data of five measurements in four groups of subjects are shown in Figure 1 and Table 2. Two-way repeated measures ANOVA showed no interaction between time and group for VAS and peak torque of knee extension (F = 0.930, *p* = 0.518, η^2^ = 0.052; F = 0.260, *p* = 0.994, η^2^ = 0.015). Further analysis of the main effect of time or group showed that VAS had a main effect on group (F = 10.574, *p* < 0.001, η^2^ = 0.134), and VAS and knee peak torque had a main effect on time (F = 118.946, *p* < 0.001, η^2^ = 0.699; F = 5.029, *p* = 0.001, η^2^ = 0.089). IL-6 and CK had interactions between group and time (F = 2.331, *p* = 0.008, η^2^ = 0.120; F = 4.608, *p* < 0.001, η^2^ = 0.212). The results of further comparison within or between groups are as follows.

**TABLE 2:** Changes in indicators of participants at different time points.

Index	Group	Baseline	Immediately	24 h	48 h	72 h
VAS (scores)	Vibration group	0.12 ± 0.05	2.11 ± 0.73	2.55 ± 0.99	1.45 ± 0.21	0.75 ± 0.11
	Kinesio group	0.11 ± 0.03	2.16 ± 0.69	2.60 ± 1.15	1.58 ± 0.23	0.83 ± 0.12
	Combined group	0.10 ± 0.08	1.90 ± 0.58	2.02 ± 1.23	1.02 ± 0.10	0.43 ± 0.19
	Control group	0.10 ± 0.06	2.60 ± 0.65	2.92 ± 1.28	2.06 ± 0.36	1.02 ± 0.16
IL-6 (pg/mL)	Vibration group	1.5 ± 0.3	2.2 ± 0.4	2.4 ± 0.5	1.8 ± 0.3	1.6 ± 0.1
	Kinesio group	1.5 ± 0.3	2.2 ± 0.6	2.3 ± 0.7	1.8 ± 0.4	1.6 ± 0.2
	Combined group	1.4 ± 0.2	2.1 ± 0.4	1.7 ± 0.4	1.6 ± 0.3	1.5 ± 0.3
	Control group	1.5 ± 0.2	2.7 ± 0.7	2.9 ± 0.7	2.1 ± 0.5	1.6 ± 0.2
CK (u/L)	Vibration group	116.4 ± 26.8	510.5 ± 122.0	380.7 ± 78.4	150.9 ± 30.6	132.6 ± 46.7
	Kinesio group	121.6 ± 33.0	515.0 ± 133.9	365.0 ± 92.3	153.0 ± 40.6	135.0 ± 31.0
	Combined group	123.7 ± 36.5	490.5 ± 162.3	268.3 ± 66.5	140.3 ± 26.2	140.2 ± 25.0
	Control group	120.3 ± 27.3	720.3 ± 180.6	460.2 ± 137.8	149.2 ± 35.8	129.8 ± 36.2
Peak torque of knee extension (Nm)	Vibration group	192.8 ± 41.3	168.2 ± 36.2	166.1 ± 20.7	173.6 ± 23.5	185.3 ± 34.3
	Kinesio group	195.0 ± 44.5	169.0 ± 29.1	168.5 ± 37.2	176.0 ± 37.8	187.5 ± 43.0
	Combined group	191.3 ± 35.0	178.4 ± 33.0	183.0 ± 29.1	186.2 ± 31.6	188.3 ± 20.7
	Control group	193.2 ± 39.2	160.1 ± 27.0	160.4 ± 13.2	177.2 ± 25.2	188.5 ± 17.8

At baseline, no significant differences in VAS, IL - 6, CK, and peak torque of knee extension were found between groups. Immediately afterward, the combined group had lower VAS scores (p = 0.015) and IL - 6 (p = 0.007) and CK levels (p = 0.001) than the control group. At 24 h, the combined group had lower VAS scores (p = 0.043) and higher peak torque of knee extension (p = 0.048) than the control group. IL - 6 and CK levels of the vibration, kinesio, and combined groups were all lower than those of the control group, with the combined group having the lowest levels. At 48 h, VAS scores and IL - 6 levels of the vibration, kinesio, and combined groups were lower than those of the control group, with the combined group having the lowest VAS scores. At 72 h, VAS scores of the vibration, kinesio, and combined groups were lower than those of the control group, with the combined group having the lowest VAS scores.

Intragroup comparisons at different time points of the vibration group, kinesio group, combined group and control group revealed that VAS and IL-6 gradually increased from baseline to the peak at 24 h and then decreased, with statistically significant differences (*p* < 0.001). CK gradually increased from baseline to the peak immediately and then decreased, with statistically significant differences (*p* < 0.001).

## Discussion

This study explored the individual and combined effects of vibration training and kinesio taping interventions on DOMS management in athletes. We determined whether the combination of vibration training and kinesio taping is more effective at alleviating the negative effects of DOMS in athletes compared with single interventions.

### Effects of vibration training

This study found that vibration training significantly reduced VAS at 24, 48 and 72 h in the participants. This result was consistent with previous studies in which vibration training (20–45 Hz, 10 min) significantly reduced VAS immediately and at 24 and 48 h in male college students (Wheeler and Jacobson, 2013), and vibration training (50 Hz, 10 min) significantly reduced VAS at 48 and 72 h in athletes (Cheng et al., 2022a). Vibration training decreases DOMS following eccentric exercise in elite hockey players (48 and 72 h) (Akehurst et al., 2021), and vibration training following eccentric exercises reduces muscle soreness perception in elite athletes (Iodice et al., 2019). When vibration training is performed at the DOMS site, muscle spindles and α-motor neurons are stimulated, causing a pain perception response with strong muscle contraction to reduce pain (Cheng et al., 2022a). Vibration training activates large-diameter fibres in muscles and inhibits small-diameter fibres, thereby reducing pain (Akehurst et al., 2021).

This study found that vibration training significantly reduced the serum IL-6 concentrations immediately and at 24 and 48 h, as well as the serum CK concentrations immediately and at 24 h, in participants. IL-6 is a potent pain-inducing inflammatory response factor and can be used as one of the important indicators of the inflammatory response (Aletaha et al., 2023). IL-6 can measure the muscle injury caused by DOMS; the higher the concentration, the stronger the acute inflammatory response and the more obvious the muscle injury (Dos Santos et al., 2020). In addition, CK mainly exists in human skeletal muscle and cardiomyocytes, providing energy for muscle contraction (Lygate, 2024), whereas muscle injury is mainly related to exercise intensity (Małkowska and Sawczuk, 2023). In this study, DOMS was modelled on the knee joints of participants, so CK mainly comes from skeletal muscle. When participants experience DOMS, muscle tissue injury and changes in the permeability of skeletal muscle cell membranes occur, and CK is abundant in the blood. The more obvious the symptoms of DOMS, the higher the CK concentration (Cheng et al., 2022a). Our previous study found that vibration training (50 Hz, 10 min) reduces the concentrations of IL-6 and CK immediately and at 24 and 48 h (Cheng et al., 2022a). Many studies have reported similar conclusions. Vibration training (30 Hz, 10 min) can reduce the concentrations of IL-6 and CK at 24 and 120 h, reducing the inflammatory response caused by DOMS (Cardinale and Bosco, 2003). Vibration training (50 Hz, 5 min) can significantly reduce the concentrations of IL-6 and CK at 48 h in young women (Imtiyaz et al., 2014). Moreover, vibration training (50 Hz, 30 min) can significantly reduce the concentrations of IL-6 and CK immediately and at 12 and 24 h in ordinary young people (Bakhtiary et al., 2006). The mechanism was explored as follows: vibration training can promote the blood and lymph circulation of the participants at the vibration site; CK in the blood rapidly enters the systemic circulation via lymph circulation, accelerates the recovery speed of the inflammatory response of DOMS and promotes the repair and remodelling process of the damaged muscle tissue (Cheng et al., 2022a; Cardinale and Bosco, 2003; Bakhtiary et al., 2006). This study showed that vibration training reduced the acute inflammatory response caused by DOMS in athletes and accelerated the recovery process of the body.

This study tested the participants’ peak torque of knee extension, showing that the vibration group gradually decreased from the baseline to the lowest value immediately and then increased, with no statistically significant differences at different time points. By contrast, the control group showed statistically significant differences. This result suggested that the vibration group could reduce the decline in muscle strength caused by DOMS in athletes. Koh et al. (2013) conducted vibration training (50 Hz, 5 min) on the elbow of ordinary young women, showing a significant increase in MVIC at 72 h. Bakhtiary et al. (2006) conducted vibration training (50 Hz, 30 min) on the quadriceps femoris of ordinary men/women, and they found a significant increase in MVIC at 24 h. We previously believed that compared with the control group, vibration training significantly increased the peak torque of knee extension in athletes (immediately and at 24, 48 and 72 h) (Cheng et al., 2022a), consistent with this study. This phenomenon may be related to the increase in muscle tension caused by vibration training, which activates additional motor units (at the DOMS site) (Cheng et al., 2022a; Koh et al., 2013; Bakhtiary et al., 2006). In view of the reduction of pain in the vibration group, participants observed a reduction in the decline in muscle strength caused by pain when performing isokinetic muscle strength tests (Cheng et al., 2022a).

### Effects of kinesio taping

This study found that kinesio taping significantly reduced the VAS of participants at 48 and 72 h. A series of studies confirmed that kinesio taping can reduce muscle pain caused by DOMS (Lee et al., 2015; Bae et al., 2014; Haksever et al., 2016; Song and Yang, 2021), and it was found to reduce the VAS of the biceps brachii in young men (Lee et al., 2015). Bae et al. (2014) supported this view that kinesio taping effectively reduces the VAS of the biceps brachii in healthy men (immediately, 24 h and 48 h). It was also effective when the participants were female and when the DOMS site was the thigh. Haksever et al. (2016) found that kinesio taping significantly reduces the VAS of the rectus femoris and hamstring muscles in young women. Similar conclusions were also reached when the participants were athletes. Song and Yang (2021) believed that kinesio taping can reduce the VAS of athletes immediately and at 24 and 48 h. These studies support the conclusions of this study. The mechanism was explored as follows: the current hypothesis is that kinesio taping inhibits pain input. Kinesio taping can provide continuous tactile and proprioceptive input, thereby inhibiting pain input and exerting a pain relief effect (Abbasi et al., 2020).

This study found that kinesio taping significantly reduced the serum IL-6 levels of participants immediately and at 24 and 48 h, as well as the serum CK levels immediately and at 24 h. These results were consistent with the findings of vibration training. The effect of kinesio taping on DOMS in ordinary male college students (Bae et al., 2014; Haksever et al., 2016) was consistent with the results of this study, showing that intramuscular adhesive tape reduced the inflammatory response in the acute stage of injury and increased the recovery rate (Bae et al., 2014; Haksever et al., 2016). Some differences were found between this study and studies in which the participants were athletes. Song and Yang (2021) believed that vibration training can significantly reduce athletes’ IL-6 levels immediately and at 24 h, as well as CK levels at different time points, but the difference was not statistically significant. However, the overall trend was consistent (kinesio taping reduces the concentration of serum IL-6 and CK in athletes). The mechanism was explored as follows: DOMS reduces the diameter of blood vessels and impairs blood flow response, causing hyperalgesia (Elkeblawy et al., 2023). The tissues at the injured site of DOMS swell, affecting blood and lymph circulation (Keriven et al., 2023; Sonkodi et al., 2023); kinesio taping can lift the skin at the sticking site, increase the tissue gap and accelerate blood and lymph circulation (CK and IL-6 in the tissue fluid enter the systemic circulation with lymph circulation, increasing the metabolic rate in the human body), and anti-inflammatory factors infiltrate into the lesions to accelerate the inflammatory response and reduce pain (Xue et al., 2023; Li et al., 2023).

At present, the changes in muscle strength caused by DOMS have been reported by few studies. An early study suggested that kinesio taping can accelerate the recovery of MVIC at the two ends of the humerus (Lee et al., 2015); at the same time, it can promote the recovery of maximum isometric contraction of the knee joint (DOMS) (Haksever et al., 2016). This study expanded on previous studies (Lee et al., 2015; Haksever et al., 2016) and suggested that the kinesio group could reduce the tendency of muscle strength decline caused by DOMS in athletes. The mechanism was explored as follows. Studies suggested that kinesio taping increases skin input and enhances neuromuscular function (Lee et al., 2015). Kinesio taping can also promote muscle activity at the site of sticking, improve muscle structure and contribute to a small increase in muscle strength (Que, 2023). In addition, kinesio taping reduces the participants’ muscle pain, which makes them more active in isokinetic muscle strength tests.

### Effects of combined intervention

Compared with a single intervention, combined intervention further significantly reduced the concentrations of IL-6 and CK at 24 h and reduced the VAS scores at 48 and 72 h. Thus, combined intervention was better at relieving the inflammatory response, muscle micro-injury and muscle pain caused by DOMS than single intervention. Compared with the control group, combined intervention significantly increased the peak torque of knee extension of participants at 24 h, whereas a single intervention did not have a similar effect, indicating that combined intervention could relieve the muscle strength decline caused by DOMS. One study reported the effect of combined intervention on muscle pain and muscle strength of badminton undergraduates at the DOMS site, which is believed to have a better effect than a single intervention (Que, 2023). The mechanisms by which vibration training and kinesio taping improve DOMS may be multifaceted and potentially complementary when used in conjunction. For vibration training, the mechanical vibrations are thought to stimulate muscle spindle fibres, which can enhance muscle activation and lead to increased blood flow and lymphatic drainage. This increased circulation may aid in the clearance of metabolic waste products and inflammatory mediators, such as CK and IL-6, which are associated with muscle damage and inflammation. Additionally, vibration training may promote the release of endorphins, providing a natural analgesic effect that can reduce pain perception. By contrast, kinesio taping is believed to facilitate lymphatic flow and reduce inflammation by lifting the skin, which may create a conducive space for lymphatic drainage. The tape also provides support to the affected muscles without restricting circulation, which can help reduce muscle soreness and promote healing.

The combined intervention of vibration training and kinesio taping has synergistic effects. The observed synergy may result from vibration-induced hyperemia enhancing lymphatic clearance facilitated by kinesio tape’s mechanical lift. The concurrent proprioceptive input from both interventions could modulate pain perception through gate-control mechanisms, while augmented nutrient delivery accelerates muscle repair processes. The notable reduction in IL - 6 and CK levels at 24 h and decreased VAS scores at 48 and 72 h with the combined intervention suggest it provides a more comprehensive DOMS management strategy than single interventions. The increase in knee joint peak torque at 24 h with the combined intervention, not seen with single interventions, further supports the hypothesis that these two methods work complementarily to enhance muscle strength and recovery. For practical implementation, combining 10-min 50 Hz vibration sessions with Y-shaped quadriceps taping could be integrated into post-training recovery protocols, particularly after eccentric-dominant activities like downhill running.

### Limitations

This study has several limitations. First, the exclusive focus on track and field athletes from a single region restricts generalizability to other sports populations and geographic cohorts. Second, the 72-h observation window precludes assessment of long-term intervention effects, while intermittent monitoring may obscure dynamic DOMS progression. Third, individual variability in DOMS susceptibility and potential gender-specific responses were not statistically analyzed. Finally, reliance on subjective pain scales without complementary objective measures (e.g., pressure pain thresholds) limits mechanistic interpretation.

## Conclusion

Vibration training and kinesio taping could alleviate muscle pain and reduce serum IL-6 and CK concentrations and muscle strength loss caused by DOMS in athletes to varying degrees. The two intervention methods had similar effects. Compared with single intervention, combined intervention was more effective in reducing inflammatory response and muscle micro-injury and reducing muscle pain and muscle strength loss.

## Data Availability

The raw data supporting the conclusion of this article will be made available by the authors, without undue reservation.
